# Differential Expression of Extracellular Matrix and Adhesion Molecules in Fetal-Origin Amniotic Epithelial Cells of Preeclamptic Pregnancy

**DOI:** 10.1371/journal.pone.0156038

**Published:** 2016-05-24

**Authors:** Myung-Sun Kim, Ji Hea Yu, Min-Young Lee, Ah Leum Kim, Mi Hyun Jo, MinGi Kim, Sung-Rae Cho, Young-Han Kim

**Affiliations:** 1 Division of Maternal Fetal Medicine, Department of Obstetrics and Gynecology, Yonsei University College of Medicine, Seoul, Korea; 2 Department and Research Institute of Rehabilitation Medicine, Severance Hospital, Seoul, Korea; 3 Institute of Women’s Life Medical Science, Yonsei University College of Medicine, Seoul, Korea; 4 Yonsei Stem Cell Research Center, Avison Biomedical Research Center, Seoul, Korea; 5 Brain Korea 21 PLUS Project for Medical Science, Yonsei University College of Medicine, Seoul, Korea; CHA University, REPUBLIC OF KOREA

## Abstract

Preeclampsia is a common disease that can occur during human pregnancy and is a leading cause of both maternal and neonatal morbidity and mortality. Inadequate trophoblast invasion and deficient remodeling of uterine spiral arteries are associated with preeclampsia (PE). The development of this syndrome is thought to be related to multiple factors. Recently, we isolated patient-specific human amniotic epithelial cells (AECs) from the placentas of 3 women with normal pregnancy and 3 with preeclamptic pregnancy. Since the characteristics of human AECs in PE are different from those in normal pregnancy, we sought to confirm the genes differentially expressed between preeclamptic pregnancy and normal pregnancy. Therefore, we performed transcriptome analysis to investigate the candidate genes associated with the possible pathophysiology of preeclampsia. Pathway analysis was performed using the Database for Annotation, Visualization, and Integrated Discovery (DAVID) and Kyoto Encyclopedia of Genes and Genomes (KEGG) online resource. In this study, we selected a total of 12 pathways and focused on extracellular matrix-related and biological adhesion molecules. Using RT-PCR array and real-time PCR, we confirmed that COL16A1, ITGB2, and LAMA3 were significantly up-regulated, but ITGA1, ITGA3, ITGA6, MMP1, MMP3, MMP10 and MMP11 were significantly down-regulated in preeclamptic fetal origin cells. Taken together, we suggest that the genes and pathways identified here may be responsible for the occurrence and development of PE, and controlling their expression may play a role in communication with fetal-maternal placenta to keep normal pregnancy.

## Introduction

Preeclampsia (PE) is a common condition in human pregnancy and is a leading cause of both maternal and neonatal morbidity and mortality [1]. It has been reported that a lot of in vitro and animal models have been used to study aspects of preeclampsia, and the most common being models of placental oxygen dysregulation, abnormal trophoblast invasion, inappropriate maternal vascular damage and anomalous maternal-fetal immune interactions [2]. Also, this syndrome is thought to be almost certainly the result of multiple biological effects including to oxidative stress and genetic factors [1].

During the developmental processes, the amnion of the placenta derived from organs epiblast of the embryo as early as 8 days after fertilization. The epiblast gives rise to the amnion as well as to all of the germ layers of the embryo [3–6]. Gastrulation occurs at approximately 3 weeks after fertilization, which is nearly 2 weeks after amniotic epithelial cells (AECs) are formed from the epiblast [3,4]. Thus, the amnion may retain the pluripotent properties of early epiblast cells, thereby allowing the AECs to retain several unique characteristics [3–6]. Therefore, these cells derived from the fetal side of the placenta may play a role in maintenance of normal pregnancy.

In order to investigate differentially expressed genes (DEGs) profiling, we performed transcriptome analysis using fetal-origin AECs from normal and preeclamptic pregnancy. The transcriptome pertains to the total set of transcripts expressed from the entire genome in a given organism, including all mRNA transcripts in the cell. The expression pattern of the transcriptome is referred to as gene expression profiling and/or, transcriptome analysis. Gene expression profiling is important for determine the cellular function or the cell-specific phenotype of a disease. It can be used to analyze the cause and the potential result of a disease. Gene expression profiling can be helpful for identifying potential drug targets.

We hypothesized that the DEGs involved in the pathophysiology of preeclampsia could be identified using this DEGs profiling by transcriptome analysis. In this study, our aim was to identify the disease-specific DEGs from transcriptome analysis and to analyze candidate genes associated with the possible pathophysiology of preeclampsia in fetal side. Therefore, we focused on extracellular matrix and adhesion molecules rather than the whole data of gene expression profiling.

## Materials and Methods

### Subjects

Human placentas were obtained from 3 healthy pregnant women and 3 pregnant women with PE. The women had live singleton births between September 2013 and July 2014 at the Department of Obstetrics and Gynecology, Severance Hospital, Yonsei University College of Medicine. The indication for a cesarean section in the healthy pregnant women was as follows: two had previously undergone cesarean section while the third had placenta previa. The diagnosis of PE was in accordance with the definitions of the American College of Obstetricians and Gynecologists. PE was defined as the presence of hypertension and proteinuria after 20 weeks of gestation. Gestational hypertension was expected when the blood pressure exceeded 140mm Hg systolic or 90mm Hg diastolic. Proteinuria was defined by 24-h urinary excretion exceeding 300mg of persistent 1+ dipstick protein in random urine samples [7]. Women with fetal structural anomaly, preterm labor, preterm rupture of membranes, cardiovascular disease, renal disease, hepatic disease, diabetes, chronic hypertension, and infectious disease were excluded in this study. The human placentas were obtained after cesarean delivery, with the approval of the Institutional Review Board of Severance Hospital, Yonsei University Health System approved this consent procedure and the entire study (no. 4-2013-0344). Participants provided their written informed consent to participate in this study, and IRBs approved this consent procedure.

### Preparation and culture of fetal-origin AECs

The amnions from normal and preeclamptic pregnancy were washed with ice-cold Hanks’ balanced salt solution (HEBB; Invitrogen Life Technologies, Carlsbad, CA) without calcium and magnesium until they were free of blood contamination. The fetal-origin smooth surface of the amniotic layer was mechanically peeled off with an autoclaved microscope slide and minced using a dissection blade. To isolate AECs, the amnion membrane was incubated with 0.05% trypsin-EDTA (Invitrogen Life Technologies) at a constant temperature of 37°C in a stirred water bath for 30 min. This step was repeated three times. The first digested supernatant was discarded. The second and third digested supernatants were collected into new tubes, and DMEM containing FBS was added. Next, these supernatants were gently centrifuged for 5 min, and the supernatant was discarded. The digested pellets were plated using Supplement Pack/Placental epithelial cell growth medium (PromoCell, Heidelberg, Germany).

### Immunocytochemistry for characterization of fetal-origin AECs

Immunocytochemistry (ICC) was performed as previously described [8]. To confirm the expression of endogenous cytokeratin-7 in AECs, cells were stained with anti-cytokeratin-7 antibody (1:400; Abcam) and incubated with Alexa Fluor^®^ 488 goat anti-rabbit (1:400, Invitrogen) secondary antibody. Samples were mounted on glass slides with fluorescent mounting medium with 4’,6-diamidino-2-phenylindole (DAPI; Vectorshield, Vector). Confocal imaging (LSM700, Zeiss, Gottingen, Germany) was used to image the cells.

### RNA preparation

Total RNA was isolated from fetal-origin AECs of PE patients and normal controls. RNA isolation was performed using Trizol (Invitrogen Life Technologies) according to the manufacturers’ protocols and stored at −80°C until further use. For quality control, RNA purity and integrity were evaluated by gel electrophoresis and the OD 260/280 ratio was determined with a Nanodrop spectrophotometer (Thermo, Waltham, MA). DNase I (Invitrogen Life Technologies) treated on total RNA before cDNA synthesis.

### RT-QPCR for characterization of fetal-origin AECs

To confirm the characterization of fetal-origin AECs, the genes expressed in AECs were analyzed by RT-QPCR. One microgram of purified total RNA was used as a template to generate the first strand cDNA using a cDNA kit (Invitrogen Life Technologies). Then, 1 μl of cDNA in a total volume of 20 μl was used for PCR. The following thermocycler parameters were used: amplifications were respectively performed followed by 35 cycles at 95°C for 30 s, 54°C for 30 s and 72°C for 30 s. Amplification was evaluated by 2% agarose gel electrophoresis. Primers used for RT-QPCR were as follow: Oct-3/4, 5’-ATCCTGGGGGTTC TATTTGG-3’ and 5’-CTCCAGGTTGCCTCTCACTC-3’, Nanog, 5’-TTCCTTCCTCCATGGAT CTG-3’ and 5’-TCTGCTGGAGGCTGAGGTAT-3’, and Sox2, 5’-AACCCCAAGATGCACAAC TC-3’ and 5’-CGGGGCCGGTATTTATAATC-3’. GAPDH was used as the internal control [3,9]. Primers of GAPDH were 5’-CAAGGTCATCCATGACAACTTTG-3’ and 5’-GTCCACCA CCCT GTT GCTGTAG-3’.

### RNA sequencing and transcriptome data analysis

RNA sequencing was performed by Macrogen Inc (Seoul, Korea). The mRNA was transcribed into a library of templates. This successive cluster generation using the reagents provided by the Illumina^®^ TruSeq^™^ RNA Sample Preparation Kit [10–12]. We performed the transcriptome analysis by follow procedures: RNA-seq experiment and data handling procedure. The detail procedures of RNA-seq experiment are performed by following the instruction. The first, there are 8 steps in TruSeq mRNA library construction: purify and fragment mRNA, synthesize first strand cDNA, synthesize second strand cDNA, perform end repair, adenylate 3' ends a single, ligate adapters, enrich DNA fragments, enriched library validation.

Purify and fragment mRNA process purifies the poly‐A containing mRNA molecules using poly‐T oligo attached magnetic beads using two rounds of purification. During the second elution of the poly‐A RNA, the RNA is also fragmented and primed for cDNA synthesis. The step of synthesize first strand cDNA reverses the cleaved RNA fragments primed with random hexamers into first strand cDNA using reverse transcriptase and random primers. Next, synthesize second strand cDNA process removes the RNA template and synthesizes a replaced strand to generate double—stranded (ds) cDNA. The perform end repair process converts the overhangs resulting from fragmentation into blunt ends, using an end repair (ERP) mix. Next, ‘A’ nucleotide is added to the 3’ ends of the blunt fragments to prevent them from ligating to one another during the adapter ligation reaction. A corresponding single ‘T’ nucleotide on the 3’ end of the adapter provides a complementary overhang for ligating the adapter to the fragment. Following ligate adapters process ligates multiple indexing adapters to the ends of the ds cDNA, preparing them for hybridization onto a flow cell. In the enrich DNA fragments process, we performed PCR to enrich those DNA fragments selectively that have adapter molecules on both ends and to amplify the amount of DNA in the library. Finally, enriched library validation perform procedures for quality control analysis on the sample library and quantification of the DNA library templates [13].

The second procedure of RNA sequencing is clustering & sequencing using the Illumina. The Illumina utilizes a unique "bridged" amplification reaction that occurs on the surface of the flow cell. A flow cell with millions of unique clusters is loaded into the HiSeq 2000 for automated cycles of extension and imaging. Solexa's sequencing-by-synthesis utilizes four proprietary nucleotides possessing reversible fluorophore and termination properties [12]. Each sequencing cycle occurs in the presence of all four nucleotides leading to higher accuracy than methods where only one nucleotide is present in the reaction mix at a time. Repeating this cycle, one base at a time, we generated a series of images each representing a single base extension at a specific cluster.

The next procedure is data handling. It contains sequence quality check and data analysis. SolexaQA is a Perl-based software package that calculates quality statistics and creates visual representations of data quality from FASTQ files generated by Illumina second-generation sequencing technology. Data analysis performed by TopHat and Cufflinks. TopHat is a fast splice junction mapper for RNA-Seq reads. It aligns RNA-Seq reads to mammalian-sized genomes using the Ultra high-throughput short read aligner Bowtie, and then analyzes the mapping results to identify splice junctions between exons. Cufflinks assembles transcripts, estimates their abundances, and tests for differential expression and regulation in RNA-Seq samples. It accepts reading of aligned RNA-Seq and assembles the alignments into a parsimonious set of transcripts. Cufflinks then estimates the relative abundances of these transcripts based on how many reads support each one, taking into account biases in the library preparation protocol. Transcripts with a fold induction ≥2 and Benjamini-Hochberg adjusted p value ≤ 0.05 were considered significant and were included in downstream analysis.

### Gene ontology and pathway analyses

Gene ontology (GO) analysis was performed with the GO classification system and using the database for annotation, visualization, and integrated discovery (DAVID) software (http://david.abcc.ncifcrf.gov/) [14–16]. The functional annotation chart and clustering were used to identify enriched GO terms for biological processes, molecular functions, cellular components, and Kyoto Encyclopedia of Genes and Genomes (KEGG) pathways.

### Validation of mRNA quantification

Total RNA was prepared as described above, and reverse transcription was performed using the RT^2^ First Strand Kit (Qiagen, Hilden, Germany) according to the manufacturer’s instructions. cDNA was amplified using the Human extracellular matrix & Adhesion molecules RT^2^ PCR Array (Qiagen). The data were analyzed using the PCR array data analysis tools (Qiagen, http://www.sabiosciences.com/dataanalysis.php).

Next, 23 genes were selected, and respective genes were performed real-time PCR for validation. Real-time PCR was performed on the StepOnePlus^™^ Real Time PCR System (Thermo) using the qPCRBIO SyGreen mix Hi-Rox (PCR biosystems), according to the manufacturer’s instructions. Thermal cycling conditions were 95°C for 10 min followed by 40 cycles of 95°C for 15 sec and 60°C for 1 min. Primers used for real-time PCR were as follow: COL6A1, 5’-CGTGCTGGACAGCTCAGAGA-3’ and 5’-TCGATGACCTTGACGACGAA-3’, COL6A2, 5’-GACAGCTCCGAGAGCATTGG-3’ and 5’-CAGCCTGTTGACCACGTTGA-3’, COL14A1, 5’-AGTGGGTTGTTGCCCAATAC-3’ and 5’-ACAGGATCACTGGCCTCTTC-3’, COL16A1, 5’-CCTTCAGCAGGTGCACATCT-3’ and 5’-GGCACCCTGCTGGTAAAATC-3’, ITGA1, 5’-GAGCCTATGATTGGAATGGA-3’ and 5’-GGTTGTGTTTCGAGGGATTA-3’, ITGA3, 5’-CTGCTCACCCCTCACTCCTT-3’ and 5’-CCAATGCTGGCCACAGATAA-3’, ITGA6, 5’-CAAGGTTCTGAGCCCAAATA-3’ and 5’-TCCTCCATGCACACTTTCTG-3’, ITGB2, 5’-AATGGAGTGACGCACAGGAA-3’ and 5’-GAAGGTGATCGGGACATTGA-3’, MMP1, 5’-G GGAGATCATCGGGACAACT-3’ and 5’-GCCTGGTTGAAAAGCATGAG-3’, MMP3, 5’-ACG AGCTGGATACCCAAGAG-3’ and 5’-ATGGCTGCATCGATTTTCCT-3’, MMP10, 5’-GCACC AATTTATTCCTCGTT-3’ and 5’-CAGTGTTGGCTGAGTGAAAG-3’, MMP11, 5’-GCCTTCTT CCCCAAGACTCA-3’ and 5’-CCCCGATAGTCCAGGTCTCA-3’, LAMA2, 5’-GTCCAGGATT CCATCAGAAA-3’ and 5’-T TGCATGCTTCACATTCAGT-3’, LAMA3, 5’-GCCTTCAGGTGA CTTTTCCA-3’ and 5’-AAGTTCCTGTTCCGCAGTTC-3’, THBS2, 5’-GGTGGCCCTCCTAAG ACAAG-3’ and 5’-CGTTTCATTTTCCGCAAAGA-3’, THBS3, 5’-GGGCATGGAAATCGTTCA GA-3’ and 5’-CACCATTGAAGGCCGTGTAT-3’, and FN1, 5’-GCACCACAGCCATCTCACA T-3’ and 5’-TCCAACGGCCTACAGAATTT-3’. For real-time PCR, human β-actin was used as the internal control. Primers of human β-actin were 5’-AATGCTTCTAGGCGGACTATGA-3’ and 5’-TTT CTGCGCAAGTTAGGTTTT-3’.

### Statistical analysis

Data were expressed as mean ± standard deviation. Using Statistical Package for Social Sciences (SPSS) version 20.0, statistical analyses were performed. Student *t* test was used for the comparison of two groups. A *P* value <0.05 was considered statistically significant.

## Results

### Characteristics of study subjects

Three patients with preeclampsia and three healthy controls were included in this study. Basic demographics and clinical information are described in Table 1.

**Table 1:** Clinical Characteristics of Participants.

	Maternal age (years)	Gestational age (weeks)	Height (cm)	PP weight (Kg)	Weight at admission (Kg)	BMI (PP/admission) (kg/m^2^)	BP at admission (mmHg)	Proteinuria	Gravida	Birth weight (g)	AS(1)	AS(5)
Normal 1	28	38	158	57	75	22.8/30.0	120/80	negative	1	3190	5	9
Normal 2	34	38	163	63	64	23.7/24.0	102/77	negative	2	3180	7	8
Normal 3	38	38	158	77	87	30.8/34.8	125/70	negative	4	3560	7	9
Preeclampsia 1	29	35	166	51	65	18.5/23.5	170/100	2+	1	1710	7	8
Preeclampsia 2	37	34	158	43	58	17.2/23.2	150/90	3+	6	2300	6	7
Preeclampsia 3	39	32	163	70	78	26.3/29.3	160/100	1+	2	1400	5	7

PP, pre-pregnant; BMI, body mass index; BP, blood pressure; AS, Apgar score

### Characterization of fetal-origin AECs

**Fig 1:**
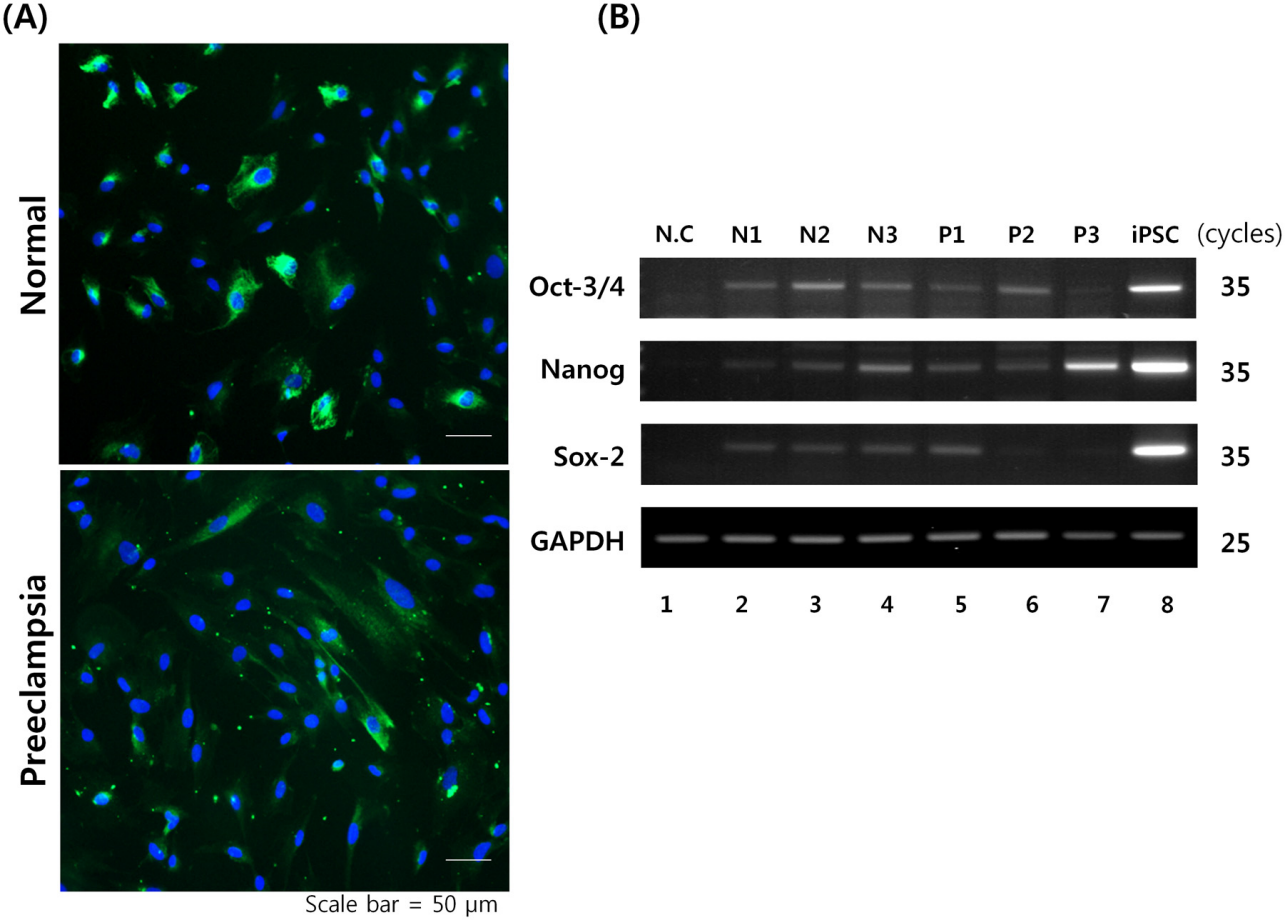
The characteristics of fetal origin AECs. Scale bar = 50μm. (A) Fetal origin AECs from a healthy pregnant woman, and a pregnant woman with PE. AECs marker cytokeratin-7 was visualized by Alexa Fluor^®^ 488 goat anti-Rabbit secondary antibody (green). And nuclei were counter stained with 4’,6-diamidino-2-phenylindole (DAPI). (B) RT-QPCR analysis was used to determine Oct4, Nanog, and Sox-2, which are indicating their stem-like potential. (lane 1, human fibroblast as a negative control; lane 2–4, normal AECs; lane 5–7, patient AECs; and lane 8, iPSC from normal AECs). RT-QPCR was performed 35 cycles, and GAPDH was used as the internal control, and performed 25 cycles.

In order to confirm the characteristics of fetal-origin AECs, we performed staining for cytokeratin-7 antibody after obtaining the placentas. The results indicated that these fetal-origin AECs expressed cytokeratin-7, which is a known marker for AECs (Fig 1A). Furthermore, fetal origin AECs had differing morphology between PE and normal pregnancy samples. Previous reports indicated that human amnion-derived cells (hADCs) are a heterogeneous group of multipotent progenitor cells that can be readily derived from placental tissue after delivery [3–6]. Our results from RT-QPCR in the fetal origin AECs indicated the stem-like potential by expression of endogenous ESC markers including Oct4, Nanog, and Sox-2, though their expressions were weaker than expression in iPSC (Fig 1B). As a negative control of these ESC markers, human skin fibroblast was used (Fig 1B, lane 1).

### Transcriptome analysis

Total RNA was prepared from samples obtained from preeclamptic patients (n = 3) and healthy controls (n = 3). We performed transcriptome analysis involving RNA sequencing in order to identify genes differentially expressed between preeclamptic patients and controls; 31,625 differentially expressed transcripts (S1 Table) were obtained. Analysis of transcripts showing 2.0-fold up- or down-regulation showed that the expression levels of 305 transcripts were lower and those of 503 transcripts were higher in preeclamptic patients than in controls (S2 Table).

### GO Consortium analysis

**Fig 2:**
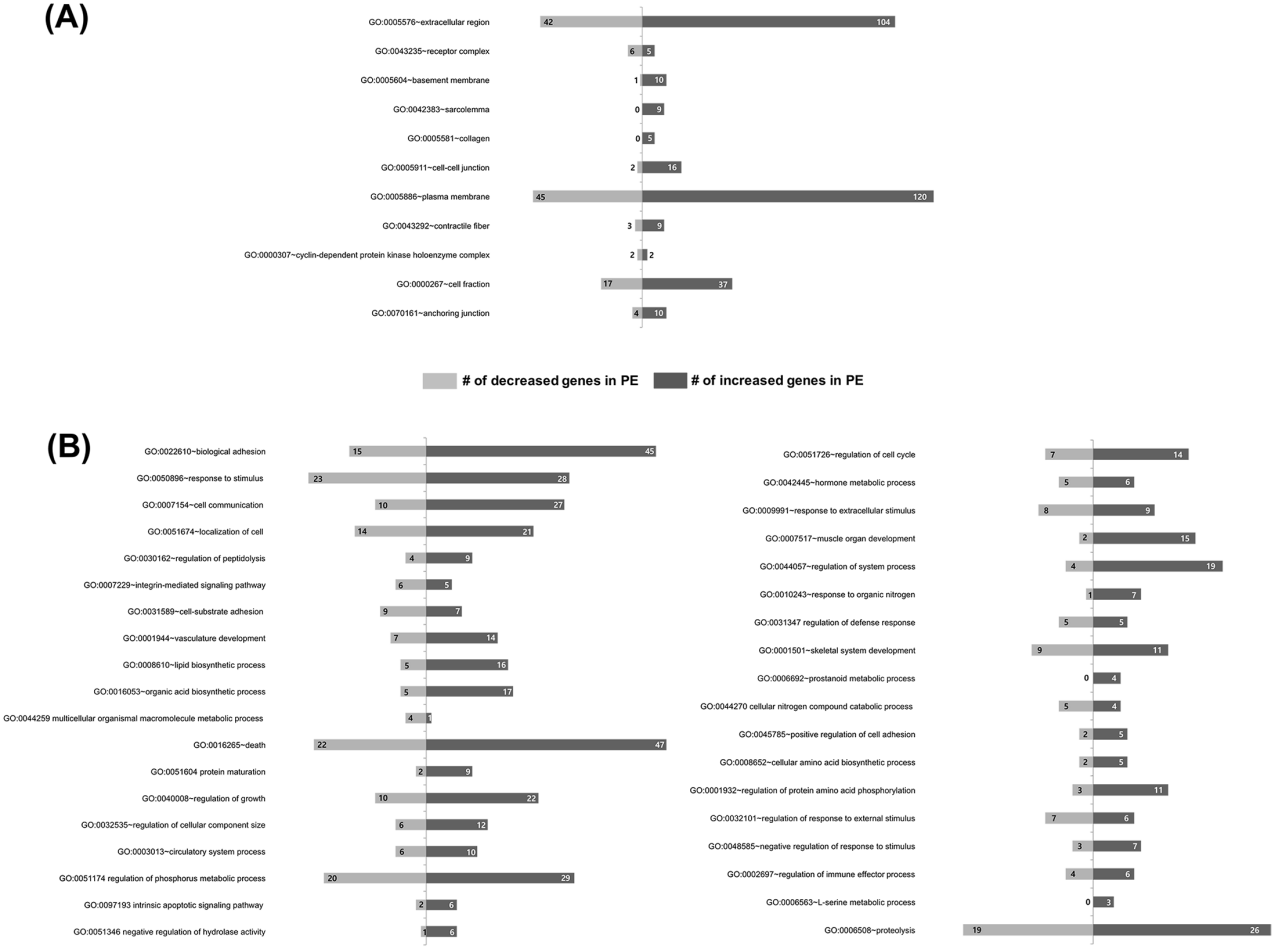
Gene-ontology analysis of DEGs between PE and normal pregnancy. (A) Main categories of increased or decreased genes in PE on the basis of their cellular components. (B) Main categories of increased or decreased genes in PE on the basis of their biological processes. The numbers indicate involved genes. (*F*-statistic *p*<0.05).

The expressed data sets were organized into GO Consortium grouping. These analyses were checked with p-values of <0.05 for the data set analyzed. For GO analysis, the cellular component (CC) and biological processes (BP) was first analyzed. This was followed by functional annotation clustering for similarity among grouping. CC classification identified the extracellular region, receptor complex, basement membrane, sarcolemma, collagen, cell-cell junction, plasma membrane, contractile fiber, cyclin-dependent protein kinase holoenzyme complex, cell fraction, and anchoring junction clustering (Fig 2A). Of these, the extracellular region and plasma membrane showed the major clustering. In extracellular region clustering, 104 genes showed increased and 42 genes showed decreased expression. Within the plasma membrane clustering, 120 genes showed increased and 45 genes showed decreased expression in PE. BP classification also showed a total of 37 clustering genes that included those involved in biological adhesion, response to stimulus, cell communication, integrin-mediated signaling, vasculature development, and cell death (Fig 2B). These results indicated that biological adhesion, response to stimulus, cell communication, integrin-mediated signaling, vasculature development, and death processes might play roles in the pathophysiology of PE.

### KEGG pathway analysis

In order to analyze pathways, total DEGs were classified based on information regarding gene function using GO from KEGG pathway database. This analysis demonstrated hsa04512:Extracellular matrix (ECM)-receptor interaction, hsa04510:focal adhesion, hsa05410:hypertrophic cardiomyopathy (HCM), hsa03320:PPAR signaling pathway, hsa04350:TGF-beta signaling pathway, hsa05412:arrhythmogenic right ventricular cardiomyopathy (ARVC), hsa05414:dilated cardiomyopathy, hsa05219:bladder cancer, hsa04115:p53 signaling pathway, hsa04514:cell adhesion molecules (CAMs), hsa04614:renin-angiotensin system, and hsa05200:pathways in cancer (Table 2) were all statistically significant (p<0.05).

**Table 2:** Pathways associated with preeclampsia subjects generated by DAVID.

Term	Count	%	P Value	Genes
hsa04512:ECM-receptor interaction	18	0.27	1.40E-07	ITGA11, ITGB4, ITGA1, ITGB5, COL5A3, SDC4, HMMR, LAMA2, LAMB3, LAMA3, ITGA6, ITGB6, COL6A3, COL6A2, COL6A1, LAMC2, THBS2, COL11A1
hsa04510:Focal adhesion	27	0.41	9.30E-07	ITGA11, ITGB4, ITGB5, LAMB3, ITGB6, COL6A3, COL6A2, COL6A1, PDGFD, THBS2, COL11A1, SHC4, FLT1, ITGA1, COL5A3, FLNB, LAMA2, LAMA3, CCND3, ITGA6, CCND2, FYN, MAPK3, VEGFA, PDGFRA, LAMC2, MAPK8
hsa05410:Hypertrophic cardiomyopathy (HCM)	13	0.20	4.00E-04	PRKAB2, ITGA11, ITGB4, ITGA1, ITGB5, TPM2, TPM4, LAMA2, TNNT2, ACE, DES, ITGA6, ITGB6
hsa03320:PPAR signaling pathway	10	0.15	0.004	OLR1, SCD, FABP3, SLC27A6, FABP4, SCD5, PCK2, ACSL3, MMP1, PLTP
hsa04350:TGF-beta signaling pathway	11	0.17	0.006	PPP2R1B, BMP2, LTBP1, CDKN2B, INHBE, ID1, MAPK3, GDF5, DCN, THBS2, BMP6
hsa05412:Arrhythmogenic right ventricular cardiomyopathy (ARVC)	10	0.15	0.007	LAMA2, DES, DSG2, ITGA6, PKP2, ITGB6, ITGA1, ITGB4, ITGA11, ITGB5
hsa05414:Dilated cardiomyopathy	11	0.17	0.008	TNNT2, LAMA2, DES, ITGA6, ITGB6, ITGA1, ITGB4, ITGA11, ITGB5, TPM2, TPM4
hsa05219:Bladder cancer	7	0.11	0.011	TYMP, CDKN1A, CDKN2A, MAPK3, VEGFA, CDH1, MMP1
hsa04115:p53 signaling pathway	9	0.14	0.011	STEAP3, CDKN1A, CDKN2A, TNFRSF10B, CCND3, CCND2, BBC3, SFN, CDK2
hsa04514:Cell adhesion molecules (CAMs)	12	0.18	0.037	F11R, CLDN7, CLDN4, PTPRF, ITGA6, CLDN6, CLDN1, CDH1, L1CAM, VCAN, CLDN11, SDC4
hsa04614:Renin-angiotensin system	4	0.06	0.039	ACE, REN, MME, ANPEP
hsa05200:Pathways in cancer	23	0.35	0.041	BMP2, WNT5B, PTGS2, PML, FOXO1, CDH1, ZBTB16, CDK2, MMP1, FZD7, LAMA2, LAMB3, CDKN1A, CDKN2A, LAMA3, CDKN2B, ITGA6, VEGFA, MAPK3, PDGFRA, LAMC2, MAPK8, WNT6

These pathways are statistically significant (P-value < 0.05)

**Fig 3:**
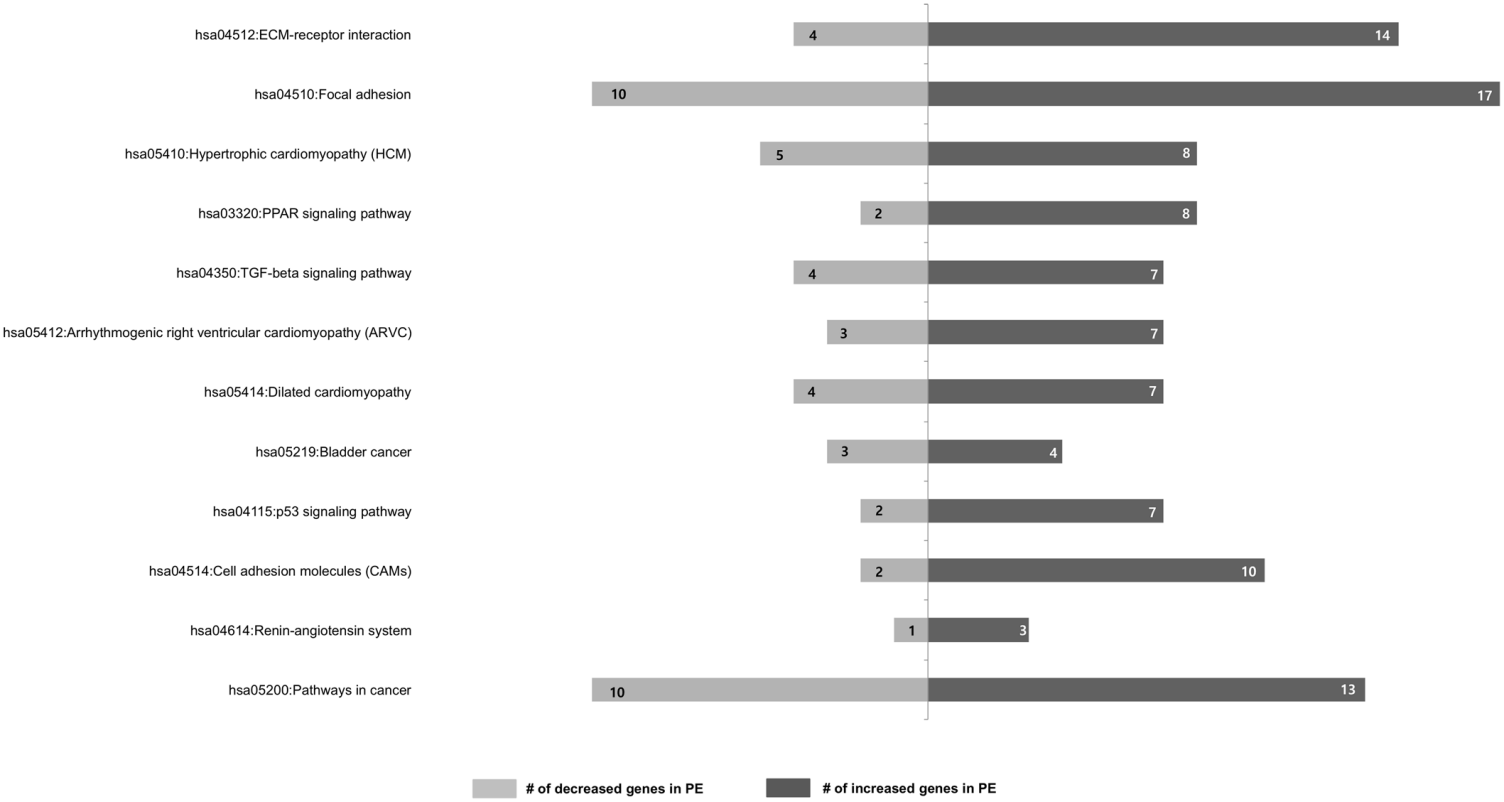
Information regarding gene function using gene ontology from KEGG pathway database. The numbers indicate involved genes. (*F*-statistic *p*<0.05).

Representatively, in hsa04512: ECM-receptor interaction, 14 genes showed increased and 4 genes showed decreased expression. In hsa04510: focal adhesion, 17 genes showed increased expression in PE (Fig 3). Since the results of this pathway analysis were similar to GO term results, our study suggests that biological adhesion, including ECM-receptor interaction and focal adhesion, might play roles in PE and normal pregnancy. When we classified up- and down-regulate genes in PE patients and investigated the results of pathway analysis, it was found that 8 pathways were significantly up-regulated and 2 pathways were significantly down-regulated in PE subjects (p<0.05) (Tables 3 and 4).

**Table 3:** Pathways upregulated in preeclampsia subjects generated by DAVID.

Term	Count	%	P Value	Genes
hsa04512:ECM-receptor interaction	14	0.34	7.83E-07	ITGA11, ITGB4, COL5A3, SDC4, LAMA2, LAMB3, LAMA3, ITGB6, COL6A3, COL6A2, COL6A1, LAMC2, COL11A1, THBS2
hsa04510:Focal adhesion	17	0.41	2.47E-04	FLT1, ITGA11, ITGB4, COL5A3, LAMA2, LAMB3, LAMA3, CCND3, FYN, ITGB6, COL6A3, COL6A2, COL6A1, LAMC2, PDGFD, COL11A1, THBS2
hsa03320:PPAR signaling pathway	8	0.19	0.004	OLR1, SCD, FABP3, SLC27A6, SCD5, PCK2, ACSL3, PLTP
hsa05410:Hypertrophic cardiomyopathy (HCM)	8	0.19	0.012	TNNT2, LAMA2, ACE, DES, ITGB6, ITGB4, ITGA11, TPM4
hsa04115:p53 signaling pathway	7	0.17	0.014	STEAP3, CDKN1A, CDKN2A, TNFRSF10B, CCND3, BBC3, SFN
hsa04514:Cell adhesion molecules (CAMs)	10	0.24	0.015	F11R, CLDN7, CLDN4, PTPRF, CLDN6, CLDN1, CDH1, L1CAM, CLDN11, SDC4
hsa05412:Arrhythmogenic right ventricular cardiomyopathy (ARVC)	7	0.17	0.024	LAMA2, DES, DSG2, PKP2, ITGB6, ITGB4, ITGA11
hsa04350:TGF-beta signaling pathway	7	0.17	0.043	LTBP1, CDKN2B, INHBE, GDF5, DCN, THBS2, BMP6

These pathways are statistically significant (P-value < 0.05)

**Table 4:** Pathways downregulated in preeclampsia subjects generated by DAVID.

Term	Count	%	P Value	Genes
hsa04510:Focal adhesion	10	0.37	0.007	ITGA6, CCND2, MAPK3, VEGFA, PDGFRA, ITGA1, ITGB5, MAPK8, FLNB, SHC4
hsa04910:Insulin signaling pathway	7	0.26	0.027	PRKAR2B, PHKG1, PRKAB2, MAPK3, FOXO1, MAPK8, SHC4

These pathways are statistically significant (P-value < 0.05)

### Validation of mRNA quantification

**Fig 4:**
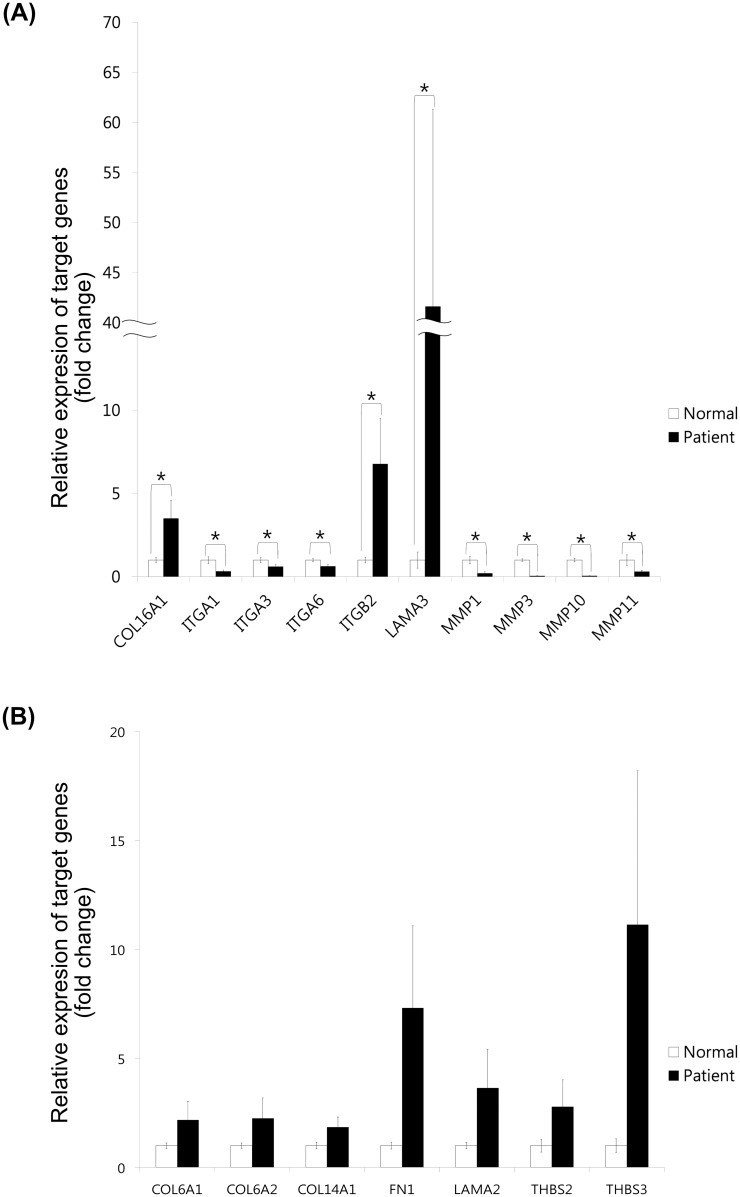
Validation for mRNA quantification of ECM-related and biological adhesion molecules. (A) The relative expression levels for COL16A1, ITGB2, and LAMA3 were significantly up-regulated, but ITGA1, ITGA3, ITGA6, MMP-1, MMP-3, MMP-10 and MMP-11 were significantly down-regulated in AEC from patients with PE. (B) COL6A1, COL6A2, COL14A1, FN1, LAMA2, THBS2, and THBS3 were up- or down-regulated, but not significant in AEC from patients with PE.

Therefore, we focused on ECM-related and biological adhesion, and performed RT-PCR array (Qiagen), since this kit included our interesting and related genes. Next, we performed validation of the majority genes on ECM-related and biological adhesion using real-time PCR (Fig 4). Expression levels of these genes in AEC from patients with PE relative to that in AEC from normal subjects as determined by real-time PCR were as follows: COL16A1 (3.50-fold, t = -2.395, *p* = 0.029), ITGB2 (6.77-fold, t = -2.220, *p* = 0.041), LAMA3 (41.62-fold, t = -2.188, *p* = 0.044), ITGA1 (0.32-fold, t = 3.383, *p* = 0.004), ITGA3 (0.60-fold, t = 2.295, *p* = 0.036), ITGA6 (0.62-fold, t = 3.057, *p* = 0.008), MMP-1 (0.20-fold, t = 3.482, *p* = 0.003), MMP-3 (0.04-fold, t = 13.160, *p*<0.001), MMP-10 (0.05-fold, t = 9.388, *p*<0.001), and MMP-11 (0.30-fold, t = 2.263, *p* = 0.038) (Fig 4A), and COL6A1 (2.18-fold, t = -1.448, *p* = 0.167), COL6A2 (2.25-fold, t = -1.399, *p* = 0.181), COL14A1 (1.84-fold, t = -1.821, *p* = 0.087), FN1 (7.31-fold, t = -1.769, *p* = 0.098), LAMA2 (3.63-fold, t = -1.561, *p* = 0.138), THBS2 (2.78-fold, t = -1.488, *p* = 0.156), and THBS3 (11.12-fold, t = -1.519, *p* = 0.148) (Fig 4B). Of them, COL16A1, ITGB2, and LAMA3 were significantly up-regulated, but ITGA1, ITGA3, ITGA6, MMP-1, MMP-3, MMP-10 and MMP-11 were significantly down-regulated in AEC from patients with PE.

## Discussion

PE is a common condition in human pregnancy and a leading cause of both maternal and neonatal morbidity and mortality [1]. The placenta is a connection tissue between the mother and fetus and plays an important role in pregnancy maintenance. Current research has mostly focused on placental tissues collected from the mother’s side of the placenta; however, there have also been significant advances in the study of the fetal side of placenta. We expected that abnormality on the fetal side of the placenta may be related to the pathophysiology and AECs in PE.

In this study, three patients with PE and three healthy controls were included, and basic demographics and clinical information were described in Table 1. Although we considered these characters of three patients with PE, preeclampsia 1 and 3 had intrauterine growth restriction (IUGR), but preeclampsia 2 had normal fetal growth (Table 1). However, in case of preeclampsia 2, the characters such as early gestational age, proteinuria, and gravida matched with PE even though fetal growth is normal (Table 1). Here, selecting the homogenous PE patients was not easy even though the gestational age between normal and PE groups were matched. In addition, we obtained AECs from three patients with PE and three healthy controls, and confirmed that the morphology of AECs in PE different from that of normal AECs (Fig 1A). Although we matched the gestational age between two groups, gestational age of 3 in healthy pregnant women was 38 weeks, but gestational age of 3 in PE was from 32 to 35 weeks. Therefore, it may be possible that the difference in characteristics of AECs between two groups is due to different gestational age. However, it has been defined that gastrulation occurs approximately 3 weeks after fertilization, which is nearly 2 weeks after AECs are formed from the epiblast [3,6]. Therefore, we suggest that the basic characteristics of AECs are due to preeclamptic pathology than different gestational age since AECs are from the early stages of human development.

It has been reported that fetal-origin AECs express endogenous Oct4, Nanog, and Sox-2, indicating their stem-like potential [3,5]. Here, we confirmed the expression of the known genes via RT-QPCR as compared to that seen for human induced pluripotent stem cells (iPSCs) (Fig 1B). DEG profiling was performed for PE and normal pregnancy patients using transcriptome analysis before the generation of patient-specific iPSCs from human AECs and their differentiation to trophoblasts.

For the functional annotation clustering, the classification stringency statistical option was set at “Medium”. From our data, within these parameters, we identified the extracellular region, the receptor complex, the basement membrane, sarcolemma, collagen, the cell-cell junction, the plasma membrane, contractile fibers, the cyclin-dependent protein kinase holoenzyme complex, cell fraction and anchoring junction clustering in CC classification (Fig 2A). Of these, since these 2 clusters have many DEGs, we selected the extracellular region and plasma membrane. BP classification showed a total of 37 clustering. These included biological adhesion, response to stimulus, cell communication, integrin-mediated signaling, vasculature development, and cell death (Fig 2B). These results indicated that biological adhesion, response to stimulus, cell communication, integrin-mediated signaling, vasculature development, and death processes might play roles in the pathophysiology of PE. The analysis of 12 pathways using the KEGG pathway database (Table 2) helped identify two pathways related to ECM-receptor interaction and focal adhesion. In hsa04512:ECM-receptor interaction, 14 genes showed increased and 4 genes showed decreased expression, and in hsa04510:focal adhesion, 17 genes showed increased expression in PE (Fig 3). Since this pathway analysis was similar to GO term results, we suggest that biological adhesion including ECM-receptor interaction and focal adhesion plays a role in PE and normal pregnancy.

We further classified the up- and down- regulated genes in the PE patients (Tables 3 and 4). At first, ECM-related and biological adhesion molecules of these genes were confirmed and validated using RT-PCR array kit, including our interesting and related genes (not shown). According to this validation, collagen related genes were up-regulated, and all MMPs were down-expressed, but TIMPs including TIMP1 and TIMP3 were up-regulated in preeclamptic fetal origin cells (not shown). It has been reported that the activity of MMPs is regulated by several types of TIMPs, and the balance between MMPs and TIMPs is largely responsible for the control of degradation of ECM proteins [17,18]. The deregulation of the balance between MMPs and TIMPs is a characteristic of diverse pathological conditions, such as rheumatoid and osteoarthritis, cancer progression, and acute and chronic cardiovascular diseases [17–21]. It has been demonstrated that human trophoblast invasiveness *in vitro* depends on the production of MMPs and that both MMP2 and MMP9 are secreted by human trophoblasts isolated from first trimester placenta [22]. Therefore, it has been reported that there were significantly reduced MMP2, and 9 expression at low oxygen level in preeclamptic patients [18]. However, it has been reported that MMP1 increased, but TIMP1 and COL1A1 decreased in the vasculature of preeclamptic women [23]. However, the results were not statistically significant since our samples were mixture. Therefore, we performed real-time PCR again for the statistically significant differences. In conclusion, we showed that COL16A1, ITGB2, and LAMA3 were significantly up-regulated, but ITGA1, ITGA3, ITGA6, MMP-1, MMP-3, MMP-10 and MMP-11 were significantly down-regulated in AEC from patients with PE (Fig 4).

It has been reported that PE is a complex pathological process that is closely related with many genes and biological processes [24]. Previous studies reported the importance of ECM–receptor interaction and focal adhesion pathways in the progression of PE. Focal adhesion pathways are activated by ECM–receptor interactions, demonstrating the importance and crosstalk of these two pathways [25,26]. Our report is demonstrating additional relevant genes here to put more importance on these two pathways.

Additionally, we related these important pathways with cell adhesion molecules. Previous reports have linked cell adhesion to immunity and inflammation [25]. These reports indicate that cell adhesion molecules mediate the attachment of leucocytes to the endothelium, which initiates endothelial dysfunction [24]. This endothelial dysfunction is a hallmark of PE [27]. Therefore, relevant genes pertaining to ECM-receptor interaction, focal adhesion, and cell adhesion molecules were obtained. The collagen VI-encoding gene was one of the genes identified. Collagen VI is a heterotrimer consisting of alpha1(VI), alpha2(VI), and alpha3(VI) chains, which are encoded by the COL6A1, COL6A2 and COL6A3 genes, respectively. In contrast to the data here, COL6A1and COL6A2 have been found to be down-regulated, while COL6A3 were found to show normal expression levels in whole chorionic villi in PE [25,28]. In addition, it has been reported that collagen V shares a number of characteristics with collagen XI [29] and hypermethylation of COL5A1 is associated with altered mRNA expression in preterm PE placentas [30]. In this study, COL5A1 and COL11A1 were up-regulated in the PE samples, suggesting that these genes may affect PE and normal pregnancy.

There are a few reports on the relationship between integrin and laminin-related genes in PE. It has been reported that ITGA5 is expressed normally in whole chorionic villi in PE [25,28]. However, our data obtained the integrin-related genes ITGA11, ITGB4, ITGB6, ITGA6, ITGA1, and ITGB5 but not ITGA5. Therefore, we suggest that it is important to study whether these genes are important in the development of PE.

In addition, it has been reported that LAMA4 expression is significantly lower in human PE placentas than in control placentas. Oxidative stress plays a vital role in controlling the expression of LAMA4 through MAPK signaling pathways, leading to a possible pathological mechanism of PE [31]. However, LAMA4 is not in our data, but LAMA2, LAMB3, LAMA3, and LAMC2 were significantly higher in human AECs from the PE fetus. Due to these opposing finding, we suggest that it is important to further study the activation mechanism of LAMA2, LAMB3, LAMA3, and LAMC2 via MAPK signaling pathways.

According to the previous report [2], we expected that abnormal trophoblast invasion of PE may be affected by expression change of DEG including to ECM-related and biological adhesion. Therefore, we suggest that the characteristics of AECs are similar with those of trophoblast, which plays a major role in development of preeclampsia. It has been reported that trophectodermal cells forming the outer most epithelial layer of the blastocyst give rise to diverse trophoblast cell types [32,33], and proliferative cytotrophoblasts (CTBs) contact the amniotic epithelium (AE) at the subsequent stage [33]. Therefore, we will perform differentiation into trophoblast of iPSC from AECs of normal and PE patients, and profiling of genes related to differentiation of trophoblast.

In this study, we focused on fetal-side AECs in PE patients, which have stem-like potential. Understanding the action mechanisms of the fetal-side in PE might be important. In further study, investigation should be expanded to elucidate mechanisms involved in controlling the development of PE by utilizing our DEGs, extracellular matrix and adhesion molecules. This study could provide valuable information for understanding unknown molecular mechanisms of PE and developing targeted therapy.

## Conclusion

We isolated patient-specific human amniotic epithelial cells (AECs) from the placentas of 3 women with normal pregnancy and 3 with preeclamptic pregnancy. Since the characteristics of human AECs in PE are different from those in normal pregnancy, we performed transcriptome analysis to investigate the candidate genes associated with the possible pathophysiology of preeclampsia. In this study, we confirmed that COL16A1, ITGB2, and LAMA3 were significantly up-regulated, but ITGA1, ITGA3, ITGA6, MMP1, MMP3, MMP10 and MMP11 were significantly down-regulated in preeclamptic fetal origin cells. Taken together, we suggest that the genes and pathways identified here may be responsible for the occurrence and development. Thus, we suggest that these extracellular matrix and adhesion molecules identified here may be responsible for the occurrence and development of PE, and controlling their expression may play a role in communication with fetal-maternal placenta to keep normal pregnancy.

## Supporting Information

S1 Table Raw data for 31,625 differentially expressed transcripts. Raw data of RNA sequencing to identify genes differentially expressed between preeclamptic patients and controls; 31,625 differentially expressed transcripts were obtained.
(XLSX)

S2 Table Raw data for 808differentially expressed transcripts. Of 31,625 differentially expressed transcripts, 808 transcripts showing 2.0-fold up- or down-regulation. The expression levels of 305 transcripts were lower and those of 503 transcripts were higher in preeclamptic patients than in controls.
(XLSX)
